# Autophagy contributes to BMP type 2 receptor degradation and development of pulmonary arterial hypertension

**DOI:** 10.1002/path.5322

**Published:** 2019-08-27

**Authors:** Maria Catalina Gomez‐Puerto, Iris van Zuijen, Christopher JZ Huang, Robert Szulcek, Xiaoke Pan, Maarten AH van Dinther, Kondababu Kurakula, Catharina C Wiesmeijer, Marie‐Jose Goumans, Harm‐Jan Bogaard, Nicholas W Morrell, Amer Ahmed Rana, Peter ten Dijke

**Affiliations:** ^1^ Department of Cell and Chemical Biology and Oncode Institute Leiden University Medical Center Leiden The Netherlands; ^2^ Department of Medicine University of Cambridge Cambridge UK; ^3^ Amsterdam UMC, Vrije Universiteit Amsterdam, Department of Pulmonary Medicine Amsterdam Cardiovascular Sciences Amsterdam The Netherlands

**Keywords:** autophagy, BMPR2, *BMPR2*^*+/−*^ iPSC‐ECs, human pulmonary artery endothelial cells (PAECs), human pulmonary artery smooth muscle cells (PASMCs), inflammation, pulmonary arterial hypertension (PAH)

## Abstract

Pulmonary arterial hypertension (PAH) is characterised by an increase in mean pulmonary arterial pressure which almost invariably leads to right heart failure and premature death. More than 70% of familial PAH and 20% of idiopathic PAH patients carry heterozygous mutations in the bone morphogenetic protein (BMP) type 2 receptor (BMPR2). However, the incomplete penetrance of *BMPR2* mutations suggests that other genetic and environmental factors contribute to the disease. In the current study, we investigate the contribution of autophagy in the degradation of BMPR2 in pulmonary vascular cells. We demonstrate that endogenous BMPR2 is degraded through the lysosome in primary human pulmonary artery endothelial (PAECs) and smooth muscle cells (PASMCs): two cell types that play a key role in the pathology of the disease. By means of an elegant HaloTag system, we show that a block in lysosomal degradation leads to increased levels of BMPR2 at the plasma membrane. In addition, pharmacological or genetic manipulations of autophagy allow us to conclude that autophagy activation contributes to BMPR2 degradation. It has to be further investigated whether the role of autophagy in the degradation of BMPR2 is direct or through the modulation of the endocytic pathway. Interestingly, using an iPSC‐derived endothelial cell model, our findings indicate that *BMPR2* heterozygosity alone is sufficient to cause an increased autophagic flux. Besides *BMPR2* heterozygosity, pro‐inflammatory cytokines also contribute to an augmented autophagy in lung vascular cells. Furthermore, we demonstrate an increase in microtubule‐associated protein 1 light chain 3 beta (MAP1LC3B) levels in lung sections from PAH induced in rats. Accordingly, pulmonary microvascular endothelial cells (MVECs) from end‐stage idiopathic PAH patients present an elevated autophagic flux. Our findings support a model in which an increased autophagic flux in PAH patients contributes to a greater decrease in BMPR2 levels. Altogether, this study sheds light on the basic mechanisms of BMPR2 degradation and highlights a crucial role for autophagy in PAH. © 2019 The Authors. *The Journal of Pathology* published by John Wiley & Sons Ltd on behalf of Pathological Society of Great Britain and Ireland.

## Introduction

Pulmonary arterial hypertension (PAH) is characterised by an increase in mean pulmonary arterial pressure (greater than 25 mmHg at rest), pulmonary capillary wedge pressure (15 mmHg), and pulmonary vascular resistance (greater than 3 Wood units) 1. The disease affects the pulmonary vasculature, which is obstructed due to adverse vascular remodelling leading to ventricular dysfunction. PAH is a rare condition with an incidence of 2–7.6 cases per million adults per year 2. Despite efforts to develop treatments leading to some improvement in symptoms and outcomes, patients still die prematurely of right heart failure 3, 4.

The classification of PAH comprises non‐hereditary or idiopathic PAH (iPAH) and hereditary PAH, which is mostly related to heterozygous germline mutations in *BMPR2* 5, 6, 7, 8. *BMPR2* encodes for the bone morphogenetic protein (BMP) type 2 receptor, which belongs to the transforming growth factor β (TGF‐β) family. It is a transmembrane serine/threonine kinase receptor, which upon BMP binding mediates the activation of intracellular Smad downstream effectors. Interestingly, 20% of iPAH patients also carry heterozygous mutations that compromise *BMPR2* function 9. Among PAH patients, those with *BMPR2* mutations develop a more severe disease with worse survival 10.

Despite the above, the incomplete penetrance of *BMPR2* mutations (20–30%) suggests that other genetic and environmental factors such as hypoxia, inflammation 11, 12, 13, 14, 15, alterations in oestrogen metabolism 16, 17, or infections 18 contribute to the disease. Previously, autophagy imbalance has been associated with PAH 19. Autophagy is a highly regulated catabolic process that involves sequestration and lysosomal degradation of cytosolic components such as dysfunctional organelles and misfolded proteins. Stress conditions including hypoxia 20, reactive oxygen species 21, inflammation 22 and DNA damage can trigger autophagy. Microtubule‐associated protein 1 light chain 3 beta‐II (MAP1LC3B‐II) is an autophagy marker and a lipidated form of MAP1LC3B‐I. It is associated with autophagosomal membranes and is fundamental for the formation of the autophagosome 23. Lee *et al* have shown an increase in MAP1LC3B‐II protein levels in lung tissue from iPAH patients compared with controls 24. However, an increase in MAP1LC3B‐II is not a measure of autophagic flux *per se*, since it can also indicate an inhibition of autophagosome clearance 23. Therefore, it remained unclear whether the increase in MAP1LC3B‐II levels in iPAH is due to autophagy activation rather than an impairment in autophagy termination. Long *et al* have also shown a relationship between autophagy and PAH pathogenesis 25. Rats suffering from the disease and treated with the lysosomal inhibitor chloroquine, which also inhibits autophagic degradation, were shown to increase BMPR2 levels 25. Interestingly, chloroquine treatment was found to prevent the development of PAH as well as its progression 25, 26.

To date, the role of autophagy in PAH remains inconclusive, as well as its role in BMPR2 degradation. In this study, we demonstrate that BMPR2 is degraded through the lysosomal pathway in an autophagy‐related fashion in primary human pulmonary artery endothelial cells (PAECs). Our data show, for the first time, that *BMPR2* heterozygosity causes an increase in autophagy contributing to the reduction in BMPR2 levels. Likewise, we demonstrate that pro‐inflammatory cytokines, which are typically elevated in PAH, are sufficient to trigger autophagy in PAECs. Finally, our results suggest an increase in autophagy in lung sections from PAH‐induced rats based on MAP1LC3B upregulation. Importantly, by means of bafilomycin A1 (BafA1) treatment, we confirmed an augmented autophagic flux in pulmonary microvascular endothelial cells (MVECs) isolated from iPAH patients.

## Materials and methods

### Antibodies and reagents

The following anti‐human antibodies were used: mouse anti‐BMPR2 (612292/clone 18; 1:500) from BD Biosciences (Vianen, The Netherlands), mouse anti‐MAP1LC3B (0231‐100/clone 5F10; 1:1000) from Nanotools (Teningen, Germany), and mouse anti‐sequestosome 1 (SQSTM1) (sc‐28359/clone D3; 1:1000) from Santa Cruz (Heidelberg, Germany). Rabbit anti‐autophagy related 7 (ATG7; 8558/clone D12B11; 1:1000) and rabbit anti‐α/β‐tubulin (2148; 1:5000) were from Cell Signaling Technology (Leiden, The Netherlands); mouse anti‐actin (A5441/clone AC‐15; 1:1000) was from Sigma‐Aldrich (Steinheim, Germany) and mouse anti‐glyceraldehyde‐3‐phosphate dehydrogenase (GAPDH; MAB374/clone 6C5; 1:10 000) was from Millipore (Amsterdam‐Zuidoost, The Netherlands). Rabbit (W4011) and mouse (W4021) horseradish peroxidase‐conjugated secondary antibodies were from Promega (Leiden, The Netherlands), and donkey anti‐mouse Alexa Fluor 488 secondary antibody (A‐21202) was from Thermo Fisher Scientific (Landmeer, The Netherlands). Hydroxychloroquine (HCQ; H0915), BafA1 (B1793), pp242 (P0037), and cycloheximide (CHX; 01810) were obtained from Sigma‐Aldrich. Rapamycin (rapa; S1039) was from Selleck Chemicals (TE Huissen, The Netherlands). Bortezomib (BTZ; SC‐217785) was from Santa Cruz and MG‐132 (474790) was from Millipore. Tumor necrosis factor alpha (TNF‐α; 300‐01A) and interleukin 1 beta (IL‐1β; 200‐01B) were obtained from PeproTech (London, UK). Autophinib (auto; 6324/10) was from Tocris Bio‐Techne (Abingdon, UK).

### Cell culture

Human dermal microvascular endothelial cells (HMEC‐1; ATCC, CRL‐3243) were cultured in MCDB131 media (10372019; Thermo Fisher Scientific) supplemented with 10% fetal bovine serum (FBS), 10 nm l‐glutamine (25030‐032; Invitrogen, Breda, The Netherlands), 1 μg/ml hydroxycortisone (H0888; Sigma), 10 ng/ml epidermal growth factor (EGF; 01‐107; Millipore), and 1 ml of penicillin (30 mg/ml)–streptomycin (48 mg/ml).

PAECs (CC‐2530) were purchased from Lonza (Basel, Switzerland) and were used between passages 4 and 8. Cells were maintained in Endothelial Cell Basal Medium 2 (C‐22211), supplemented with ECGM‐2 SupplementPack (C‐39211) from PromoCell (Heidelberg, Germany).

Human pulmonary artery smooth muscle cells (PASMCs) were purchased from Lonza (CC‐2581) and were used between passages 4 and 8. Cells were cultured in SmBM‐2 Smooth Muscle Basal medium (CC‐3181; Lonza) supplemented with SmGM‐2 Smooth Muscle SingleQuot kit (CC‐4149; Lonza). Wild‐type and *BMPR2* ^*+/−*^ iPSC‐ECs (generated and differentiated as previously described 27) were maintained in Endothelial Cell Growth Medium MV 2 (c‐22221; PromoCell) supplemented with 2% FBS, 1/100 Chemically‐Defined Lipid Concentrate, 50 μg/ml ascorbic acid, 15 μg/ml transferrin, 7 μg/ml rh‐insulin, 5 ng/ml FGF2, 20 ng/ml rhVEGF‐A_165_, 10 μm SB 431542, and 5 μm DAPT. Pulmonary MVECs were obtained from end‐stage PAH patients and healthy tissues of lobectomy donors (Table 1), as described before 28. The tissue harvesting and MVEC isolation were approved by the IRB of the VU University Medical Center (VUmc, Amsterdam, The Netherlands) and informed consent was given. Pulmonary MVECs were cultured in complete ECM medium supplemented with 1% pen/strep, 1% endothelial cell growth supplement, and 5% FCS (ScienceCell, Uden, The Netherlands). Cell lines were routinely tested for mycoplasma contamination and used only if negative.

**Table 1:** Characteristics of controls and iPAH patients

	No	mPAP (mm Hg)	Treatment	Sex	Age (years)	Surgery
	1	NA	NA	F	47	Lob
Controls	2	NA	NA	M	89	Lob
	3	NA	NA	M	69	Lob
Idiopathic PAH patients	1	43	PDE5‐I, PGI2	F	42	Ltx
	2	102	PDE5‐I, ERA, PGI2	M	21	Ltx
	3	47	PDE5‐I, ERA	F	64	Obd
	4	89	PDE5‐I, ERA, PGI2	F	22	Ltx

mPAP, mean pulmonary artery pressure; NA, not available; PDE5‐I, phosphodiesterase type 5 inhibitor; PGI2, prostacyclin; ERA, endothelin receptor antagonist; Lob, lobectomy; Obd, obduction; Ltx, lung transplantation.

### Cell line generation

To generate an HMEC‐1 line expressing Halo‐BMPR2, HMEC‐1 cells were transduced with a lentiviral plasmid with puromycin resistance carrying HaloTag‐*BMPR2* driven by an *elongation factor1* (*EF1*)*‐α* promoter. Cells were maintained in MCDB131 media, as was the parental cell line, and in the presence of 1.5 μg/ml puromycin (p7255; Sigma‐Aldrich).

### siRNA transfections

PAECs were transfected with 10 nm of human autophagy related 5 (*ATG5*; L‐004374‐00‐0005), *ATG7* (L‐020112‐00‐0005) or *BMPR2* (L‐005309‐00) RNAi (Thermo Scientific) using Lipofectamine RNAiMAX (13778075; Invitrogen). siRNA/Lipofectamine RNAiMAX complexes were allowed to form for 20 min at room temperature before being added to the cells. The media were changed 24 h after transfection. After 48 h, cells were treated and cell lysates were obtained. Knockdown efficiency was confirmed by immunoblotting or mRNA expression.

### Cell imaging

#### HaloTag staining

HMEC‐1‐HaloTag‐BMPR2 cells were treated overnight with BafA1 (20 nm). Next, they were incubated in MCDB131 media containing the non‐permeable HaloTag Alexa Fluor 488 ligand (G1001; Promega) at a dilution of 1:10 × 10^3^ for 15 min at 37°C. Cells were then washed three times with medium and images were recorded on a Leica DMi1 microscope.

#### Cyto‐ID staining

Wild‐type and *BMPR2* ^*+/−*^ iPSC‐ECs were incubated in Endothelial Cell Growth Medium MV 2 (c‐22221; PromoCell) (supplemented as described previously above) with Cyto‐ID Autophagy Detection dye (ENZ‐51031‐0050; Enzo Life Sciences, Brussels, Belgium) at a dilution of 1:500 for 25 min at 37°C. Subsequently, cells were washed and images were recorded using a Leica (Wetzlar, Germany) DMi8 microscope.

### Immunofluorescence microscopy

Rat lung tissues were fixed and stained as previously described 29. In brief, paraffin sections were deparaffinised and rehydrated. Sections were boiled for 40 min in Vector® Antigen Unmasking Solution (Vector) using a pressure cooker. After blocking with 1% BSA in 0.1% PBS, sections were incubated overnight at 4°C with primary antibodies directed against MAP1LC3B (1:50) and PECAM1 (AF3628; 1:1000) from R&D (Wiesbaden‐Nordenstadt, Germany). All sections were mounted with ProLong® Gold antifade reagent (Invitrogen) containing DAPI to counterstain the nuclei.

### Flow cytometry analysis

HMEC‐1‐HaloTag‐BMPR2 cells were treated overnight with pp242 (1 μm). Cells were then trypsinised and incubated in MCDB131 media containing the non‐permeable HaloTag Alexa Fluor 488 ligand (G100; Promega) at a dilution of 1:2000 for 15 min at 37°C. Subsequently, cells were washed twice with medium and analysed by flow cytometry. All data were analysed using FlowJo software (BD).

### Western blot quantification

Films were scanned and saved as .tiff files at 300 dots per inch (dpi). Single protein bands were quantified using ImageJ software. The resulting peaks represent the relative density of the respective lanes. Background signals were subtracted from the final quantification by drawing a baseline on the bottom of each peak. The numbers obtained for the measured area under the peak have arbitrary units and can only be compared within each particular blot. The same procedure was followed for the loading controls. The relative quantity of protein with respect to the loading control was then calculated.

### Array scan imaging

PAECs were plated in a 96‐well plate and treated with BafA1 (20 nm), TNF‐α (30 ng/ml), and IL‐1β (10 ng/ml) overnight. Cells were then washed twice with PBS and fixed with cold methanol for 10 min at −20°C. Subsequently, cells were washed twice with PBS and incubated for 1 h at room temperature with MAP1LC3B or SQSTM1 antibody at a dilution of 1:50 in PBS with 1% BSA. Cells were then washed with PBS containing 1% BSA and incubated for 1 h with donkey anti‐mouse Alexa Fluor 488 secondary antibody at a dilution of 1:250 in PBS with 1% BSA. Next, cells were washed twice with PBS and stained with Hoechst 33342 (2 μm) (62249; Thermo Fisher Scientific) for 10 min at room temperature. Sixty images per well were captured in both Hoechst and Alexa Fluor 488 channels on a Cellomics ArrayScan VTI (Thermo Scientific) using a 20 × 0.45 NA lens. Images were analysed using the Cellomics Spot Detector V4 Bioapplication (Thermo Scientific). Autofocusing was carried out on Hoechst‐stained nuclei. Exposure times were fixed for each individual experiment. Each nucleus was identified as a primary object in the Hoechst channel. MAP1LC3B and SQSTM1 spots were detected within a 24‐pixel mask defined around the nucleus. The ‘spot total intensity per object’ was reported and used to determine changes in MAP1LC3B and SQSTM1.

### RT‐qPCR

Total RNA was isolated using a NucleoSpin RNA kit (740955; Macherey Nagel, Duren, Germany) according to the manufacturer's protocol. RNA was reverse‐transcribed using a RevertAid RT Reverse Transcription Kit (K1691; Thermo Fisher Scientific). The generated cDNA was amplified with primer pairs for the indicated gene, using the CFX Connect Real‐Time PCR Detection System (Bio‐Rad). *GAPDH* was used as the reference transcript. Quantification was performed relative to the levels of *GAPDH* and normalised to control conditions. The data analysis was performed using the 2^−ΔΔCt^ method. Primer sequences are listed in the supplementary material, Table S1.

### Statistical analysis

Unpaired two‐sided Student's *t*‐tests were used to calculate statistical differences. A *P* value of less than 0.05 was considered statistically significant.

## Results

### Inhibition of lysosomal degradation results in increased BMPR2 levels in primary human pulmonary artery endothelial cells

**Figure 1:**
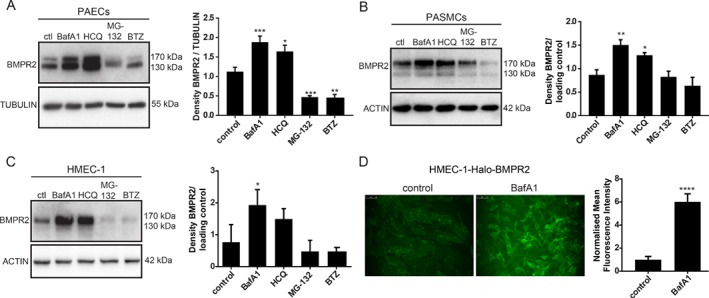
BMPR2 is degraded through the lysosomal pathway in human PAECs, PASMCs, and HMEC‐1. (A–C) BMPR2 protein levels increased after treatment with lysosomal inhibitors. Left panel: western blot analysis of BMPR2 protein levels after (A) PAECs, (B) PASMCs, and (C) HMEC‐1 were treated for 6 h with BafA1(20 nm), HCQ (20 μm), MG‐132 (5 μm), and BTZ (10 nm). Tubulin or actin was used as a loading control. Representative results of at least three independent experiments are shown. Right panels: quantification of BMPR2 levels normalised to the loading control is presented as mean ± SEM. *p < 0.05; **p < 0.005; ***p < 0.001. (D) Cell surface expression of BMPR2 after inhibition of lysosomal degradation. HMEC‐1‐Halo‐BMPR2 were treated with BafA1 (20 nm) for 16 h and stained with the non‐permeable Halo Alexa Fluor 488 ligand. Left panel: Fluorescence microscopy images of BMPR2 localisation are shown. Images are representative of at least three independent experiments. Right panel: quantification of HMEC‐1‐Halo‐BMPR2 mean fluorescence intensity is shown. Data are presented as mean ± SEM. ****p < 0.0001.

Although BMPR2 degradation has been studied before, there is still discrepancy in the results as both proteasomal degradation 30 and lysosomal 31 degradation of BMPR2 have been reported in different cell types. To investigate the mechanisms by which endogenous BMPR2 is degraded in the context of PAH, PAECs were treated with the lysosomal inhibitors BafA1 and HCQ and with the proteasome inhibitors MG‐132 and BTZ. BMPR2 levels significantly increased after treatment with lysosomal inhibitors, while treatment with proteasome inhibitors did not result in BMPR2 accumulation (Figure 1A). To validate the specificity of the antibody, *BMPR2* was knocked down using siRNA and shRNA. As expected, there was a prominent reduction in BMPR2 expression after knockdown (supplementary material, Figure S1). To confirm that the increase in BMPR2 protein levels was due to a block in lysosomal degradation rather than an increase in mRNA levels, RT‐qPCR analysis of *BMPR2* was performed in PAECs after BafA1 treatment (supplementary material, **Figure** S2). No significant differences were observed, indicating that BMPR2 accumulation occurs in response to inhibition of lysosomal degradation. To establish whether lysosomal degradation of BMPR2 is cell type‐dependent, PASMCs and HMEC‐1 (previously immortalised by others 32) were also treated with the different inhibitors (Figure 1B,C). As expected, accumulation of BMPR2 was only detected after treatment with BafA1 and HCQ (Figure 1B,C). To elucidate whether the increase in BMPR2 occurs at the plasma membrane, HMEC‐1 stably expressing *BMPR2* coupled to an N‐terminal Halo tag (Halo‐BMPR2) (supplementary material, **Figure** S3 **A**) were generated. BMPR2 overexpression and increased BMP signalling were verified by western blotting (supplementary material, **Figure** S3 **B**). The Halo tag allows for measurement of the BMPR2 protein level at the cell surface, using a non‐cell‐permeable Halo fluorescent ligand. By means of fluorescence microscopy and this ligand, the amount of Halo‐BMPR2 was measured after BafA1 treatment. The amount of Halo‐BMPR2 strongly increased upon BafA1 treatment when compared with untreated cells (Figure 1D).

Taken together, these data indicate that endogenous BMPR2 is degraded through the lysosomal pathway and that inhibition of lysosomal degradation results in BMPR2 accumulation at the plasma membrane.

### Autophagy modulation affects BMPR2 lysosomal degradation

**Figure 2:**
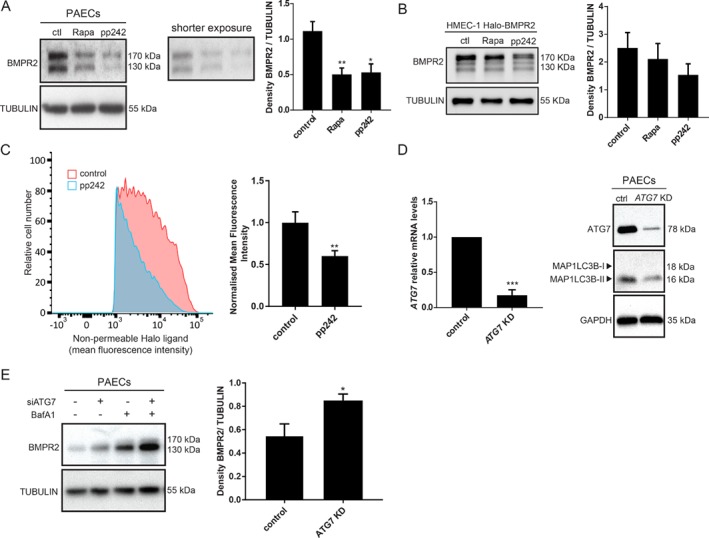
BMPR2 is degraded via lysosomes in an autophagy‐related fashion. (A, B) Western blot analysis of BMPR2 protein levels after autophagy activation. Left panel: (A) PAECs and (B) HMEC‐1‐Halo‐BMPR2 were treated for 6 h with rapa (10 μm) and pp242 (1 μm). Tubulin was used as a loading control. Representative results of at least three independent experiments are shown. Right panels: quantification of BMPR2 levels normalised to tubulin is presented as mean ± SEM. *p < 0.05; **p < 0.005. BMPR2 protein levels at the plasma membrane decrease after autophagy activation. (C) Flow cytometry‐based analysis of Halo‐BMPR2 by means of the non‐permeable Halo Alexa Fluor 488 ligand after HMEC‐1‐Halo‐BMPR2 were treated with pp242 (1 μm) for 16 h. Left panel: flow cytometry plots. Right panel: quantification of the fold Alexa Fluor 488 ligand mean fluorescence intensity upon pp242 (1 μm) treatment. The data are presented as fold increases relative to untreated control. Data of four independent experiments are presented as mean ± SEM. **p < 0.005. (D) *ATG7* knockdown efficacy was examined. Left panel: *ATG7* mRNA expression was analysed by RT‐qPCR. Data of three independent experiments performed in duplicates are presented as mean ± SEM. ***p < 0.001, normalised to *GAPDH*. The data are presented as fold increases relative to the control. Right panel: western blot showing ATG7 and MAP1LC3B protein expression. GAPDH was used as a loading control. Representative results of at least three independent experiments are shown. (E) Left panel: western blot showing BMPR2 protein levels after blocking autophagy. PAECs were transfected with control or *ATG7* siRNA followed by 6 h BafA1 treatment. Tubulin was used as a loading control. Representative results of at least three independent experiments are shown. Right panel: quantification of BMPR2 levels normalised to the loading control is presented as mean ± SEM. *p < 0.05.

Two major vesicular processes convening on the lysosome are endocytosis and autophagy 33. Importantly, chloroquine and HCQ have been found to exert beneficial effects in experimental PAH, possibly by modulating autophagy and lysosomal degradation 25. To investigate the contribution of autophagy in the pathogenesis of PAH, we explored whether autophagy modulation affects BMPR2 lysosomal degradation in endothelial cells. Human PAECs and HMEC‐1 Halo‐BMPR2 were treated with the autophagy inducers rapa 34 and pp242 35 and changes in BMPR2 protein expression were detected by western blot (Figure 2A,B). Autophagy activation resulted in a significant decrease in BMPR2 protein levels, suggesting a relationship between autophagy stimulation and BMPR2 degradation (Figure 2A,B). Similar results were obtained after starvation*‐*induced autophagy (supplementary material, **Figure** S4 **A**). The amounts of BMPR2 protein at the plasma membrane were analysed by quantifying Halo‐BMPR2 fluorescence intensity using flow cytometry (Figure 2C). As expected, HMEC‐1 Halo‐BMPR2 treated with pp242 showed a decrease in mean fluorescence intensity, compared with the control. To further confirm the role of autophagy in BMPR2 degradation, *ATG7*, a critical component regulating the elongation and closure of the phagophore membrane 36 was knocked down in PAECs by means of siRNAs. mRNA and protein levels of ATG7 were analysed, confirming its depletion (Figure 2D). In addition, to confirm a reduction in autophagy after ATG7 downregulation, the levels of the autophagy marker MAP1LC3B‐II were measured by western blot (Figure 2D). Since *ATG7* knockdown does not abrogate autophagy completely, cells were treated with BafA1 to impair the remaining autophagic flux after ATG7 depletion. Besides being an inhibitor of V‐type ATPase and certain P‐type ATPases preventing the acidification of the lysosome, BafA1 also blocks the fusion of autophagosomes with lysosomes 23, 37. Importantly, *ATG7*‐knockdown cells showed an increase in BMPR2 levels both in the presence and in the absence of BafA1 (Figure 2E). As ATG7 has autophagy‐independent roles 38, to further validate our findings, ATG5 required for MAP1LC3B‐I lipidation was knocked down in PAECs (supplementary material, **Figure** S4 **C**). Subsequently, cells were treated with CHX to stop protein synthesis excluding *de novo* BMPR2 protein production. Likewise, a block in autophagy after ATG5 depletion resulted in increased BMPR2 protein levels independently of CHX treatment (supplementary material, **Figure** S4 **C**). Taken together, these findings show that autophagy modulation in human PAECs affects BMPR2 lysosomal degradation.

### *BMPR2* heterozygosity and inflammation are sufficient to cause an increase in autophagy in endothelial cells

**Figure 3:**
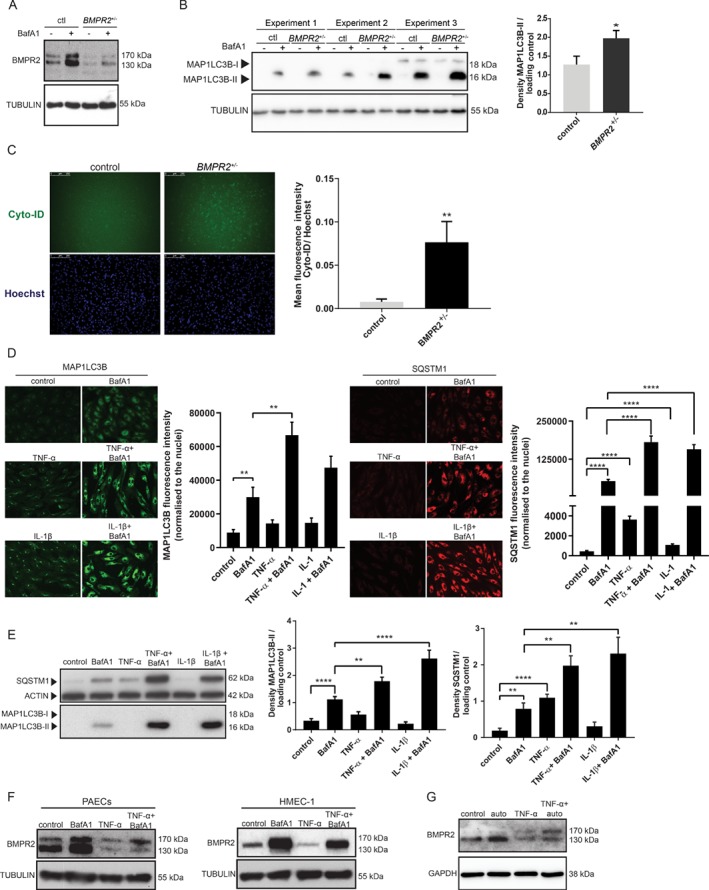
W9X mutation in *BMPR2* and inflammation are sufficient to induce autophagy in endothelial cells. (A) Western blot showing BMPR2 protein expression after *BMPR2* ^*+/−*^ iPSC‐ECs and isogenic control iPSC‐ECs were treated for 6 h with BafA1(20 nm). Tubulin was used as a loading control. Representative results of three independent experiments are shown. (B) Left panel: western blot of three independent experiments showing MAP1LC3B protein expression after *BMPR2* ^*+/−*^ iPSC‐ECs and control iPSC‐ECs were treated for 6 h with BafA1 (20 nm). Tubulin was used as a loading control. Quantification of MAP1LC3B levels normalised to the loading control is presented as mean ± SEM. *p < 0.05. Right panel: western blot showing SQSTM1 protein expression after *BMPR2* ^*+/−*^ iPSC‐ECs and control iPSC‐ECs were treated for 6 h with BafA1 (20 nm). Actin was used as a loading control. Representative results of three independent experiments are shown. Quantification of SQSTM1 levels normalised to the loading control is presented as mean ± SEM. (C) Autophagic vesicles were quantified using Cyto‐ID. Left panel: representative fluorescence microscopy images. Right panel: quantification of the Cyto‐ID mean fluorescence intensity. The data are presented as fold increases relative to Hoechst. Data of three independent experiments are shown as mean ± SEM. **p < 0.005. (D, E) PAECs were treated with TNF‐α (30 ng/ml) and IL‐1β (10 ng/ml) for 16 h or 24 h with and without BafA1 (20 nm). (D) Cells were fixed and stained for MAP1LC3B and SQSTM1. MAP1LC3B and SQSTM1 were analysed by fluorescence microscopy (array scan). Left panel: representative images showing MAP1LC3B staining in green. Array scan quantification based on ‘spot total intensity per object’. Quantification of data from three independent experiments performed in duplicates is shown as mean ± SEM. **p < 0.005. Right panel: representative images showing SQSTM1 staining in red. Array scan quantification based on ‘spot total intensity per object’. Quantification of data from three independent experiments performed in duplicates is shown as mean ± SEM. ****p < 0.0001. (E) Left panel: cells were lysed and MAP1LC3B and SQSTM1 levels were analysed by western blotting. Actin was used as a loading control. Representative results of three independent experiments are shown. Right panel: quantification of MAP1LC3B and SQSTM1 levels normalised to the loading control is presented as mean ± SEM. **p < 0.005; ****p < 0.0001. (F, G) BMPR2 levels were analysed by western blotting after cells were stimulated with TNF‐α (30 ng/ml) and BafA1 (20 nm) or auto (0.1 μm), respectively.

More than 70% of patients with hereditary PAH show heterozygous mutations that compromise *BMPR2* function. Additionally, patients with such mutations present a more severe disease 10. We therefore evaluated whether *BMPR2* heterozygosity could result in an increased autophagy contributing to the reduction of BMPR2 levels. As a model, *BMPR2* ^*+/−*^‐induced pluripotent stem cells (iPSCs) carrying a known causal *BMPR2* mutation (W9X) and an otherwise isogenic wild‐type iPSC line were differentiated towards ECs and sorted using magnetic microbeads conjugated with human PECAM1/CD31 and CDH5/CD144 antibodies 27. A reduction in BMPR2 expression in *BMPR2* ^*+/−*^ iPSC‐ECs, when compared with control iPSC‐ECs, was confirmed by western blotting analysis (Figure 3A). Subsequently, autophagic turnover was studied by analysing the levels of MAP1LC3B‐II. The level of MAP1LC3B‐II is not a measure of autophagic flux *per se*, as it can also indicate an inhibition of autophagosome clearance 23. *BMPR2* ^*+/−*^ iPSC‐ECs were therefore treated with BafA1 to prevent lysosomal degradation and to block the fusion of autophagosomes with lysosomes. Independently of the inter‐experimental differences in MAP1LC3B‐II levels, MAP1LC3B‐II was upregulated in the presence of BafA1 in *BMPR2* ^*+/−*^ iPSC‐ECs, compared with control iPSC‐ECs, indicating an increase in autophagic flux (Figure 3B). To validate our findings, autophagy changes were measured by fluorescence microscopy using Cyto‐ID, a dye selectively labelling autophagic vacuoles. *BMPR2* ^*+/−*^ iPSC‐ECs showed an increase in autophagic vacuoles, compared with control iPSC‐ECs (Figure 3C). Taken together, these data indicate that *BMPR2* heterozygosity is sufficient to cause an increase in autophagy in ECs.

An incomplete penetrance of *BMPR2* mutations suggests that other genetic and environmental factors contribute to PAH. Since inflammation is a hallmark of the disease 11, 13, we further investigated whether inflammatory cytokines previously found to be upregulated in PAH 11, 14 would trigger autophagy in PAECs. MAP1LC3B and SQSTM1, an autophagy adaptor protein whose levels are used as a reporter of autophagy activity 39, 40 were analysed by fluorescence microscopy after PAECs were treated with the pro‐inflammatory cytokines TNF‐α 39 or IL‐1β 15 in the presence or absence of BafA1 (Figure 3D). Cells treated with pro‐inflammatory cytokines showed a marked increase in MAP1LC3B and SQSTM1‐positive puncta, indicating increased formation of autophagosomes (Figure 3D). To further validate our findings, MAP1LC3B‐II and SQSTM1 levels were measured by western blotting analysis (Figure 3E). As expected, there was an accumulation in both MAP1LC3B‐II and SQSTM1 protein levels after TNF‐α and IL‐1β treatment in the presence of BafA1, compared with cells treated with BafA1 alone (Figure 3E). Our results demonstrate that inflammation increases autophagic flux in PAECs. TNF‐α has been shown to reduce BMPR2 transcription in PAECs and PASMCs 11. To investigate whether TNF‐α‐induced autophagy contributes to a decrease in BMPR2, PAECs and HMEC‐1 were stimulated with TNF‐α and BafA1, which impairs autophagy completion by inhibiting the fusion of the autophagosome with the lysosome (Figure 3F). Moreover, PAECs were treated with TNF‐α in the presence of autophinib (auto), a novel autophagy inhibitor targeting VPS34 40 (Figure 3G). As expected, TNF‐α treatment resulted in reduced BMPR2 levels. However, when cells were treated with BafA1 (Figure 3F**)** or auto (Figure 3G) together with TNF‐α, the decrease in BMPR2 levels, when compared with TNF‐α alone, was less prominent.

### Autophagy flux is increased in idiopathic pulmonary arterial hypertension

**Figure 4:**
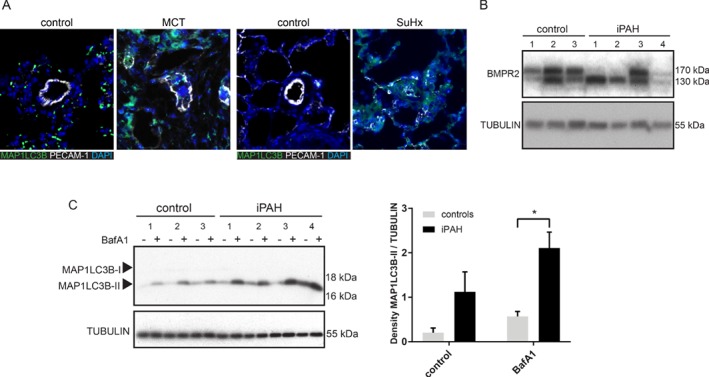
Increased autophagy is a hallmark of PAH. (A) Lung sections from MCT, SuHx, and control rats were immunostained for MAP1LC3B. Representative pictures showing MAP1LC3B staining in green, DAPI‐positive nuclei in blue, and platelet endothelial cell adhesion molecule (PECAM‐1) in white. (B) Western blot showing BMPR2 protein expression of MVECs from iPAH patients and controls. Tubulin was used as a loading control. (C) Left panel: western blot showing MAP1LC3B and SQSTM1 protein expression. MVECs from iPAH patients and controls were treated for 16 h with BafA1 (20 nm). Tubulin was used as a loading control. Right panel: quantification of MAP1LC3B and SQSTM1 levels normalised to tubulin is presented as mean ± SEM. *p < 0.05.

To investigate whether autophagy is upregulated in PAH, the levels of MAP1LC3B were determined by immunofluorescence in lung samples from two PAH rat models: monocrotaline (MCT) and SU5416*/*hypoxia (SuHx) 41. Reduced BMP signalling and BMPR2 levels have been shown in MCT and SuHx models 41. In control rats, MAP1LC3B was present at a low level, while in PAH‐induced rats, MAP1LC3B levels increased prominently (Figure 4A), possibly indicating an increase in autophagy. The representative control image for the MCT rat model shows a green fluorescence signal different to the green signal in MCT‐treated rats. Therefore, in the supplementary material, **Figure** S5, we show how this unspecific signal is also visible in the red channel. The signal seems to correspond to autofluorescent erythrocytes. This is probably due to the improper perfusion of the vessels before fixing the tissue. As mentioned before, an accumulation in MAP1LC3B may indicate a block in downstream steps of autophagy rather than an increase in the autophagic flux 45. To further elucidate whether autophagy is upregulated in PAH, pulmonary MVECs obtained from end‐stage idiopathic PAH patients (iPAH) and healthy tissues of lobectomy donors (control) (Table 1) were used (Figure 4B). iPAH patients did not harbour mutations in the *BMPR2* gene. Remarkably, a reduction in the upper band of BMPR2, which corresponds to the fully glycosylated mature form of the receptor 42, 46, was observed in three out of four iPAH MVECs compared with control MVECs (Figure 4B). *N*‐Glycosylation of BMPR2 has been shown to enhance its ability to bind BMP2 ligand 42. Interestingly, the N126 glycosylation site has been found mutated in patients with hereditary PAH 43.

Next, autophagic flux was analysed after control and iPAH MVECs were treated with BafA1, resulting in an augmented BafA1‐induced accumulation of MAP1LC3B‐II (Figure 4C). The reduction in the upper band of BMPR2 in iPAH MVECs corresponds to an increase in MAP1LC3B‐II. We are presently unable to explain the significance of this result. Taken together, these data indicate an augmented autophagy flux as a prominent characteristic of PAH.

## Discussion

We have demonstrated that endogenous BMPR2 is mainly degraded through the lysosome in PAECs and PASMCs, two cell types that play a key role in the pathology of the disease. Satow *et al* have shown that BMPR2 is degraded via the proteasomal pathway in HEK 293T cells when it is associated with Dullard phosphatase 30. In contrast, we have shown that in lung vascular endothelial cells, treatment with proteasome inhibitors resulted in a slight reduction of BMPR2 levels. This could be explained by a known induction of autophagy after treatment with proteasome inhibitors 44, 47, 48. An increase in MAP1LC3B‐II protein levels after BTZ and MG‐132 treatment in PAECs further validates this hypothesis (supplementary material, **Figure** S6 **A**). The discrepancy between the studies could be due to the comparison between endogenous and overexpressed BMPR2 degradation. The different outcomes also suggest more than one mechanism for BMPR2 degradation depending on the cell type.

Treatment with pharmacological activators of autophagy, as well as ATG7 and ATG5 depletion, confirms that in lung vascular endothelial cells, autophagy modulation affects BMPR2 lysosomal degradation. It is worth mentioning that the two lysosomal inhibitors used, BafA1 and HCQ, are also known to block autophagy by impairing autophagosome fusion with lysosomes. The exact way in which autophagy contributes to BMPR2 degradation still has to be elucidated as it could be direct or through modulation of the endocytic pathway.

Here, we have demonstrated for the first time that *BMPR2* heterozygosity is sufficient to cause an increase in autophagy. The transcript for the W9X mutation in the iPSC is degraded by nonsense‐mediated decay; therefore, the increased autophagy flux is not a response to misfolded protein accumulation. However, we cannot exclude that autophagy is triggered to clear dysfunctional mitochondria present in *BMPR2* ^*+/−*^ iPSC‐ECs 27. Furthermore, it would be interesting to understand the role of BMPR2 signalling in the regulation of autophagy and how corruption of the BMPR2 pathway contributes to its activation. As BMP9 and BMP10 circulate in the blood and selectively bind to BMPR2, we wondered whether the activation of this pathway orchestrates a negative regulation of autophagy 49, 50. Our findings show a decrease in MAP1LC3B‐II after PAECs were treated with BMP9 and BafA1, compared with BafA1 alone (supplementary material, **Figure** S7 **A**). In this way, the binding of BMP9 to BMPR2 results in a blockage of autophagy that may inhibit BMPR2 degradation, promoting its signalling.

Our data suggest that an increase in autophagy is present in lung sections from experimental rat models based on MAP1LC3B upregulation. In addition, in the same way as Lee *et al* showed an increase in MAP1LC3B‐II protein levels in lung tissue from iPAH patients 24, we demonstrated an increase in MAP1LC3B‐II in pulmonary MVECs from iPAH patients. However, by looking at the levels of MAP1LC3B‐II after BafA1 treatment, we do conclude that this is due to an increased autophagic flux.

Both *BMPR2* ^*+/−*^ iPSC‐ECs and MVECs from iPAH patients show an increased autophagy flux. Nevertheless, while SQSTM1 levels were augmented in *BMPR2* ^*+/−*^ iPSC‐ECs when compared with control iPSC‐ECs, the expression of SQSTM1 in iPAH MVECs diminished compared with control MVECs (data not shown). Although autophagy induction often corresponds to a decrease in SQSTM1 levels, SQSTM1 may be transcriptionally upregulated under certain conditions 23. In addition, a transient increase in the amount of SQSTM1 has been shown in some situations where there is an increase in autophagic flux 23. Finally, SQSTM1 has been found to exert autophagy‐independent roles that might contribute to the contradictory results observed 51.

Besides *BMPR2* mutations, insults such as inflammation, chronic hypoxia, and shear stress have been found to be involved in the pathogenesis of PAH. We demonstrated that the pro‐inflammatory cytokines TNF‐α and IL‐1β, previously found to be implicated in PAH 15, 39, 52, trigger autophagy in PAECs. Hurst *et al* reported that TNF‐α reduces BMPR2 transcription in PAECs 11. In the present study, we showed that TNF‐α‐induced autophagy also contributes to a decrease in BMPR2 levels. One of the presumed mechanisms on how inflammation induces autophagy might be through ERK1/2 MAP kinase pathway activation 28, 53. In addition, TNF‐α has also been shown to upregulate the expression of the autophagy genes *LC3* and *beclin 1* through Jun kinase signalling pathway activation and AKT/PKB inhibition 22.

Both hereditary and idiopathic forms of PAH frequently display reduced BMPR2 levels either due to mutations in *BMPR2* 10, inflammation 13 or infections 18. Our findings support a model in which an increased autophagic flux in PAH patients contributes to a greater decrease in BMPR2 levels. It will be of interest to identify adaptor proteins involved in BMPR2 degradation as well as specific ubiquitin ligases and post‐translational modifications which orchestrate this process.

Although our study reveals the possibility of inhibiting autophagy as a treatment for PAH, specific autophagy inhibitors have to be discovered and characterised. A remaining question is whether autophagy is one of the initial causes of PAH or whether it is a cellular response to counteract stress conditions that could eventually contribute to the severity of the disease. Altogether, our study sheds light on the basic mechanisms of BMPR2 degradation and highlights the crucial role of autophagy in PAH.

## Author contributions statement

MCGP and PTD conceived the project. MCGP, AAR, and PTD designed the experiments. MCGP, IVZ, CJZH, XP, and MAHVD performed experiments. MCGP, RS, and KK collected and/or assembled data and samples. MCGP, HJB, MJG, AAR, NWM, and PTD interpreted and/or analysed the data. MCGP wrote the manuscript. AAR, NWM, and PTD corrected and critically revised the manuscript. HJB, AAR, NWM, and PTD gave financial and administrative support. All authors revised the article for important intellectual content and provided final approval prior to submission for publication.

**SUPPLEMENTARY MATERIAL ONLINE**

**Figure S1.** BMPR2 antibody validation

**Figure S2.** Inhibition of lysosomal degradation does not affect *BMPR2* mRNA expression

**Figure S3.** Characterisation of HMEC‐1‐Halo‐BMPR2

**Figure S4.** Autophagy modulation results in changes in BMPR2 levels

**Figure S5.** Unspecific signal observed in the red channel

**Figure S6.** Proteasome blockage results in increased autophagy

**Figure S7.** BMP9 inhibits autophagy in human PAECs

**Table S1.** List of primers used for RT‐qPCR

## Supporting information


**Figure S1.** BMPR2 antibody validation
**Figure S2.** Inhibition of lysosomal degradation does not affect *BMPR2* mRNA expression
**Figure S3.** Characterisation of HMEC‐1‐Halo‐BMPR2
**Figure S4.** Autophagy modulation results in changes in BMPR2 levels
**Figure S5.** Unspecific signal observed in the red channel
**Figure S6.** Proteasome blockage results in increased autophagy
**Figure S7.** BMP9 inhibits autophagy in human PAECs

**Table S1.** List of primers used for RT‐qPCR
