# Supplementary material for: Autophagy contributes to BMP type 2 receptor degradation and development of pulmonary arterial hypertension
Source: J Pathol. 2019 Aug 27;249(3):356–67. doi: 10.1002/path.5322 (PMC6852495; doi:10.1002/path.5322)
Supplement: Supplementary file 1 — Figure S1. BMPR2 antibody validation Figure S2. Inhibition of lysosomal degradation does not affect BMPR2 mRNA expression Figure S3. Characterisation of HMEC‐1‐Halo‐BMPR2 Figure S4. Autophagy modulation results in changes in BMPR2 levels Figure S5. Unspecific signal observed in the red channel Figure S6. Proteasome blockage results in increased autophagy Figure S7. BMP9 inhibits autophagy in human PAECs [file PATH-249-356-s001.docx]

**Autophagy contributes to BMP type 2 receptor degradation and development of pulmonary arterial hypertension**

Gomez-Puerto MC *et al*. *J Pathol* DOI: 10.1002/path.5322

**Supplementary figures and legends**


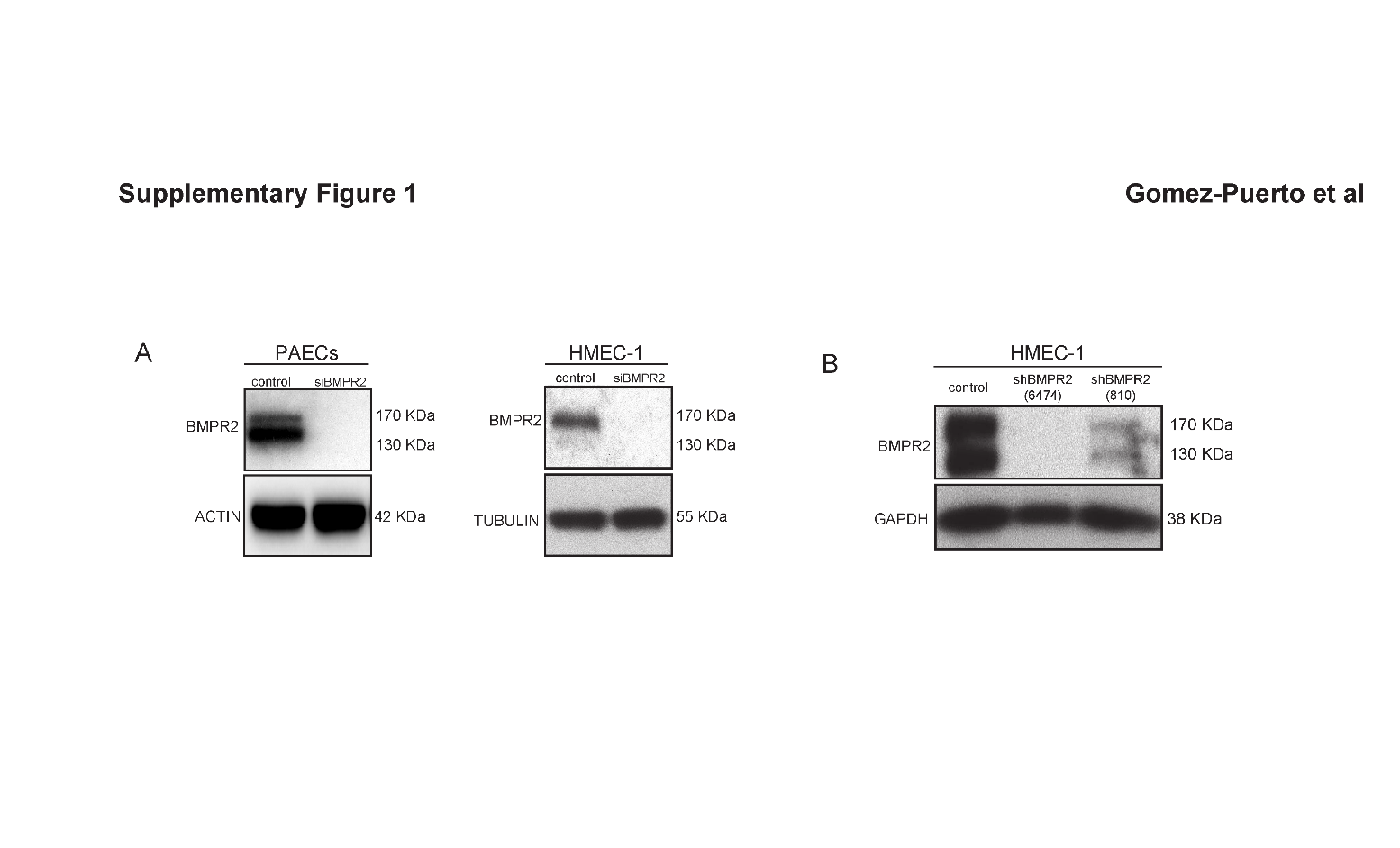


**Figure S1.** BMPR2 antibody validation. (**A**) Human PAECs and HMEC-1 were transfected with control or *BMPR2* siRNA. Cell lysates were analysed by western blotting with the indicated antibodies. Tubulin or actin was used as a loading control. Representative results of three independent experiments are shown. (**B**) HMEC-1 cells were transduced with short hairpin RNA targeting *BMPR2* and cell lysates were subjected to western blot analysis. GAPDH was used as a loading control. Representative results of three independent experiments are shown.


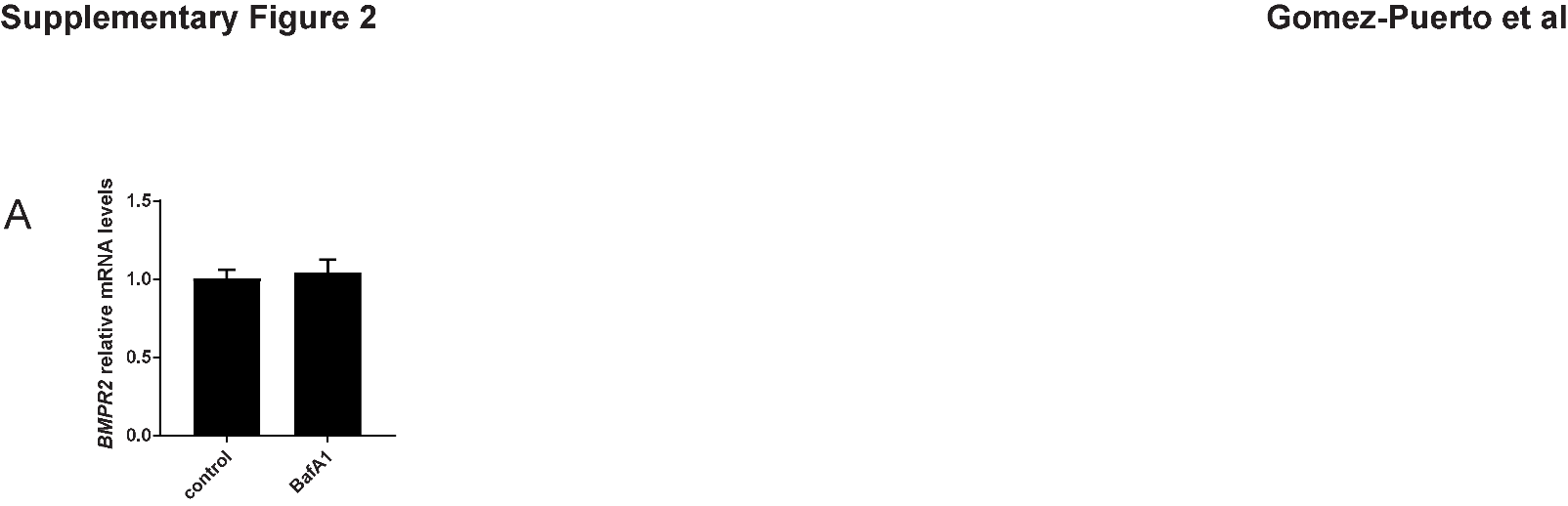


**Figure S2.** Inhibition of lysosomal degradation does not affect *BMPR2* mRNA expression. (**A**) Human PAECs were treated with BafA1 (20 nm) for 6 h and analysed for *BMPR2* mRNA expression using RT-qPCR. Data of three independent experiments performed in duplicates are presented as mean ± SEM normalised to *GAPDH*. The data are presented as fold increases relative to the control.


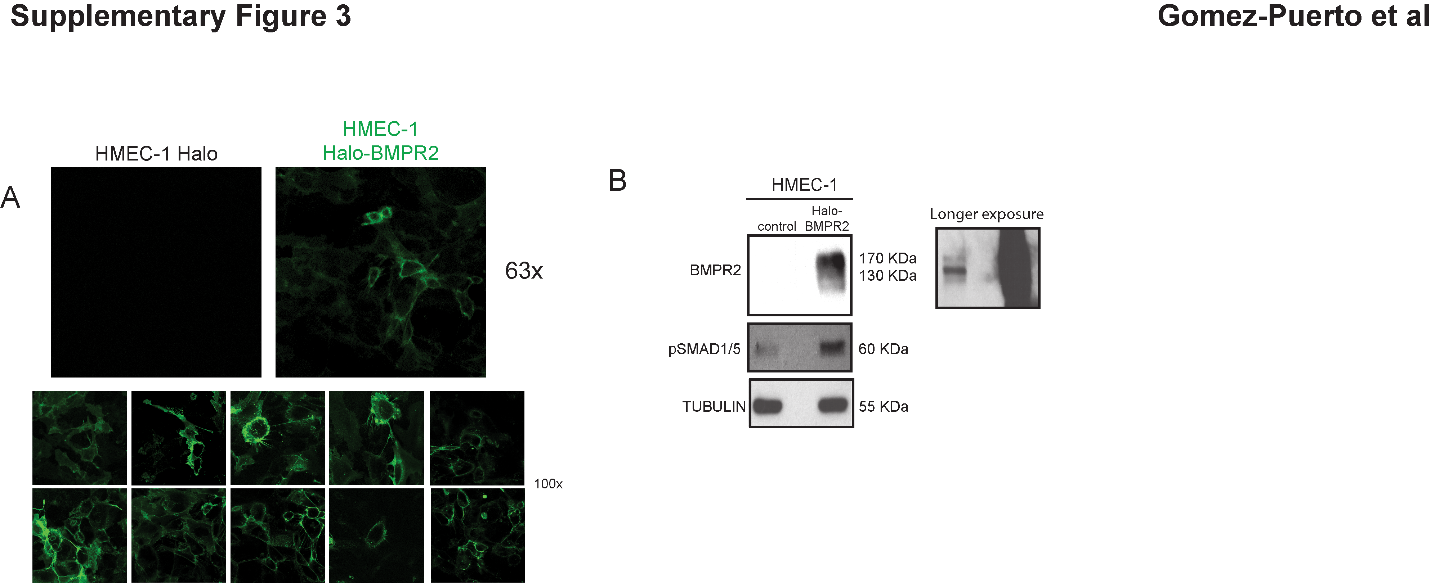


**Figure S3.** Characterisation of HMEC-1-Halo-BMPR2. (**A**) HMEC-1-Halo-BMPR2 cells were stained with the non-permeable Halo Alexa Fluor 488 ligand. The cell surface localisation of Halo-BMPR2 was confirmed by fluorescence microscopy. (**B**) BMPR2 overexpression and increased BMP signalling were confirmed by analysing BMPR2 and pSMAD1/5 protein levels by western blotting. Tubulin was used as a loading control. Representative results of two independent experiments are shown.


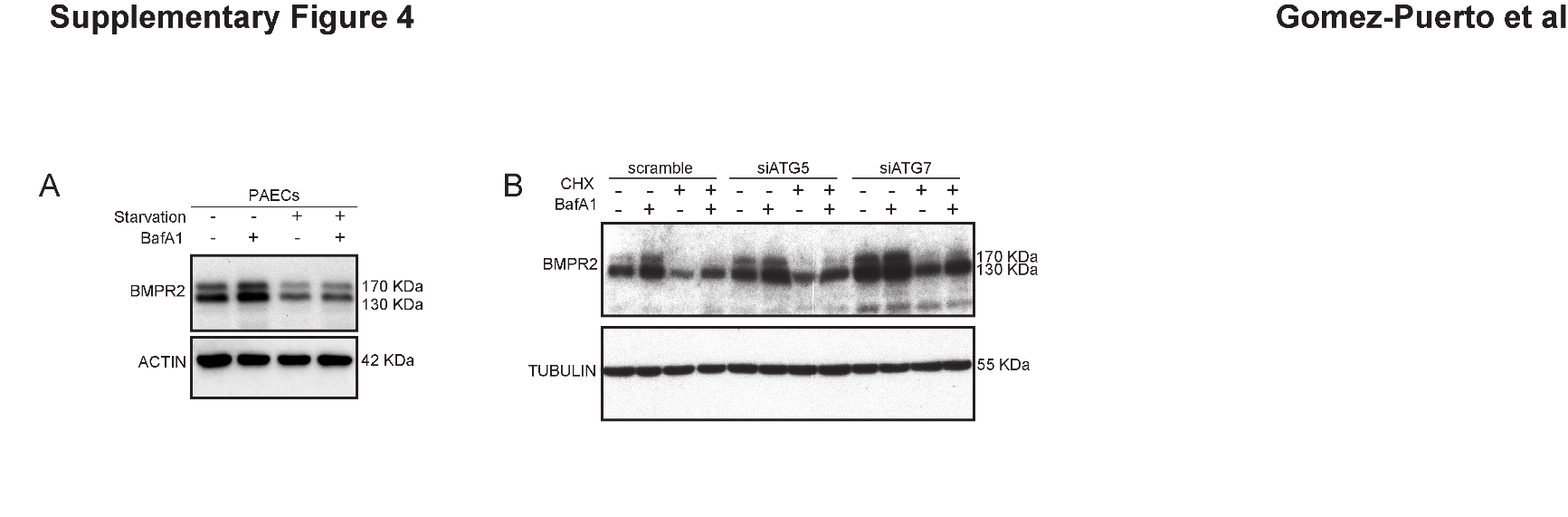


**Figure S4.** Autophagy modulation results in changes in BMPR2 levels. (**A**) Western blotting analysis of BMPR2 after human PAECs were cultured under normal or starvation conditions (0.1% FBS) for 6 h in the presence or absence of BafA1 (20 nm). Actin was used as a loading control. (**B**) Western blotting analysis of BMPR2 protein levels after blocking autophagy. PAECs were transfected with control, *ATG5* or *ATG7* siRNA followed by 2 h treatment with BafA1, CHX or BafA1 in combination with CHX. Tubulin was used as a loading control. Representative results of at least three independent experiments are shown.


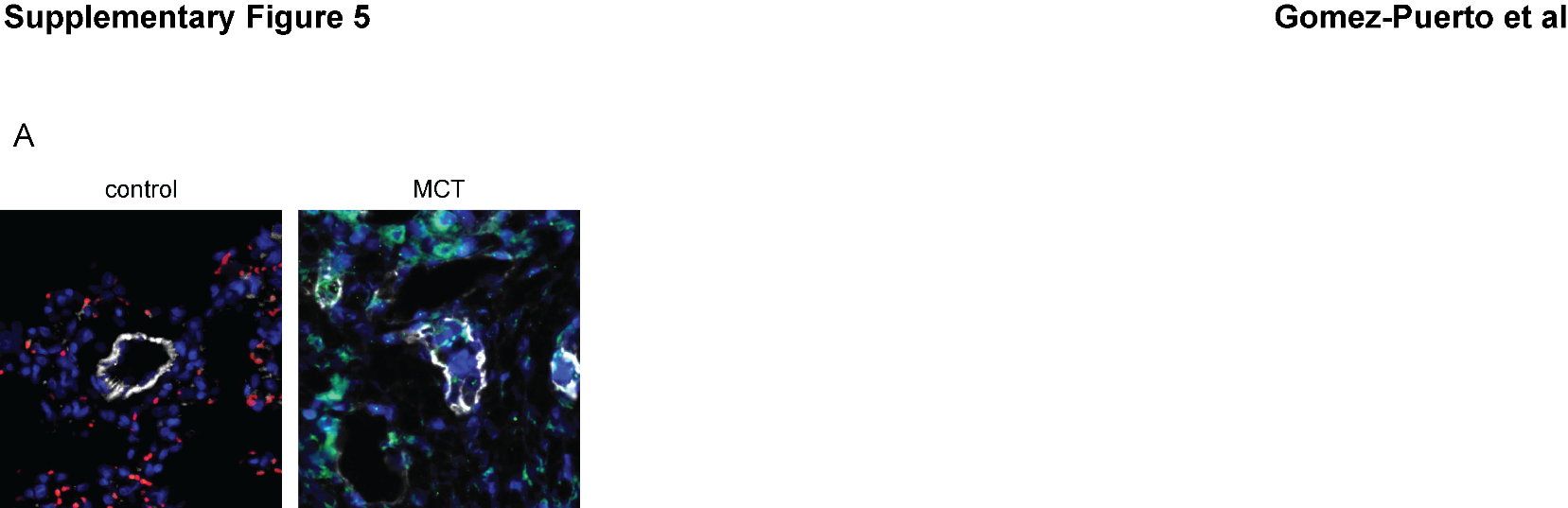


**Figure S5.** Unspecific signal observed in the red channel. The same images are shown in Figure 4A. (**A**) Lung sections from MCT and control rats were immunostained for MAP1LC3B. Representative pictures showing autofluorescence erythrocytes in red, MAP1LC3B staining in green, DAPI-positive nuclei in blue, and platelet endothelial cell adhesion molecule (PECAM-1) in white.

**
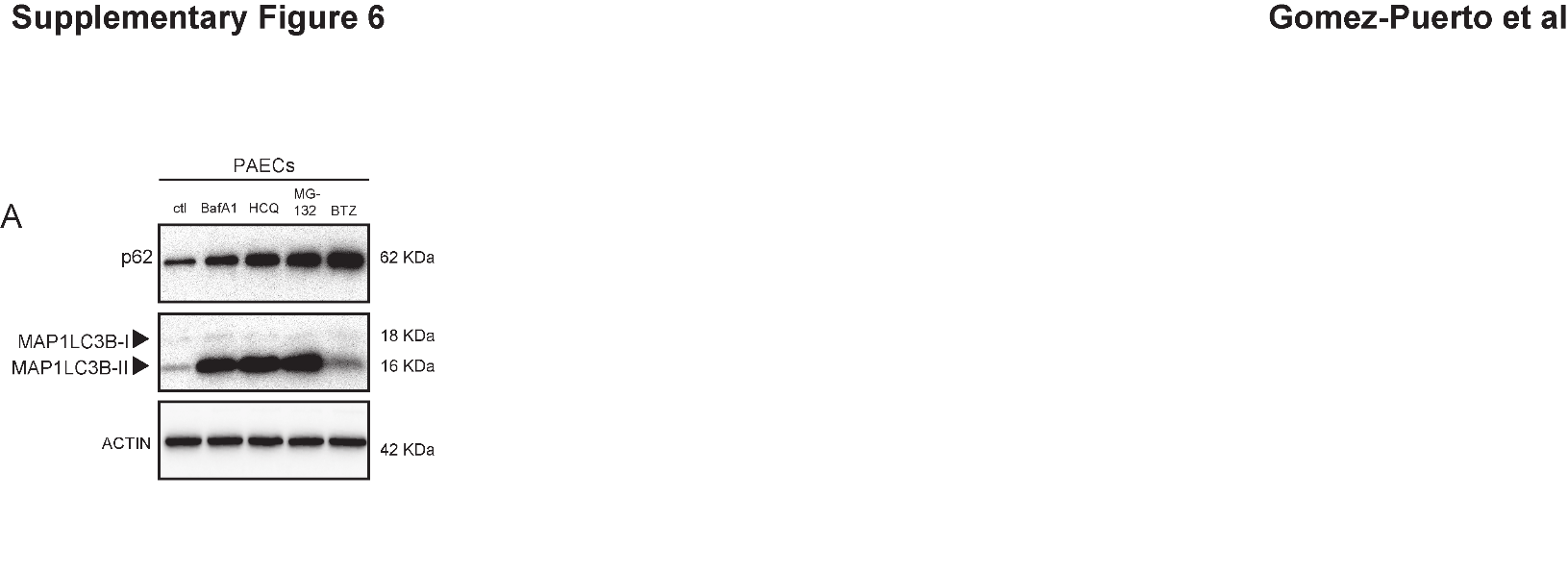
**

**Figure S6.** Proteasome blockage results in increased autophagy. (**A**) Western blotting analysis of SQSTM1 and MAP1LC3B protein expression in PAECs treated for 6 h with BafA1 (20 nm), HCQ (20 µm), MG-132 (5 µm) or BTZ (10 nm). Actin was used as a loading control. Representative results of at least three independent experiments are shown.

**
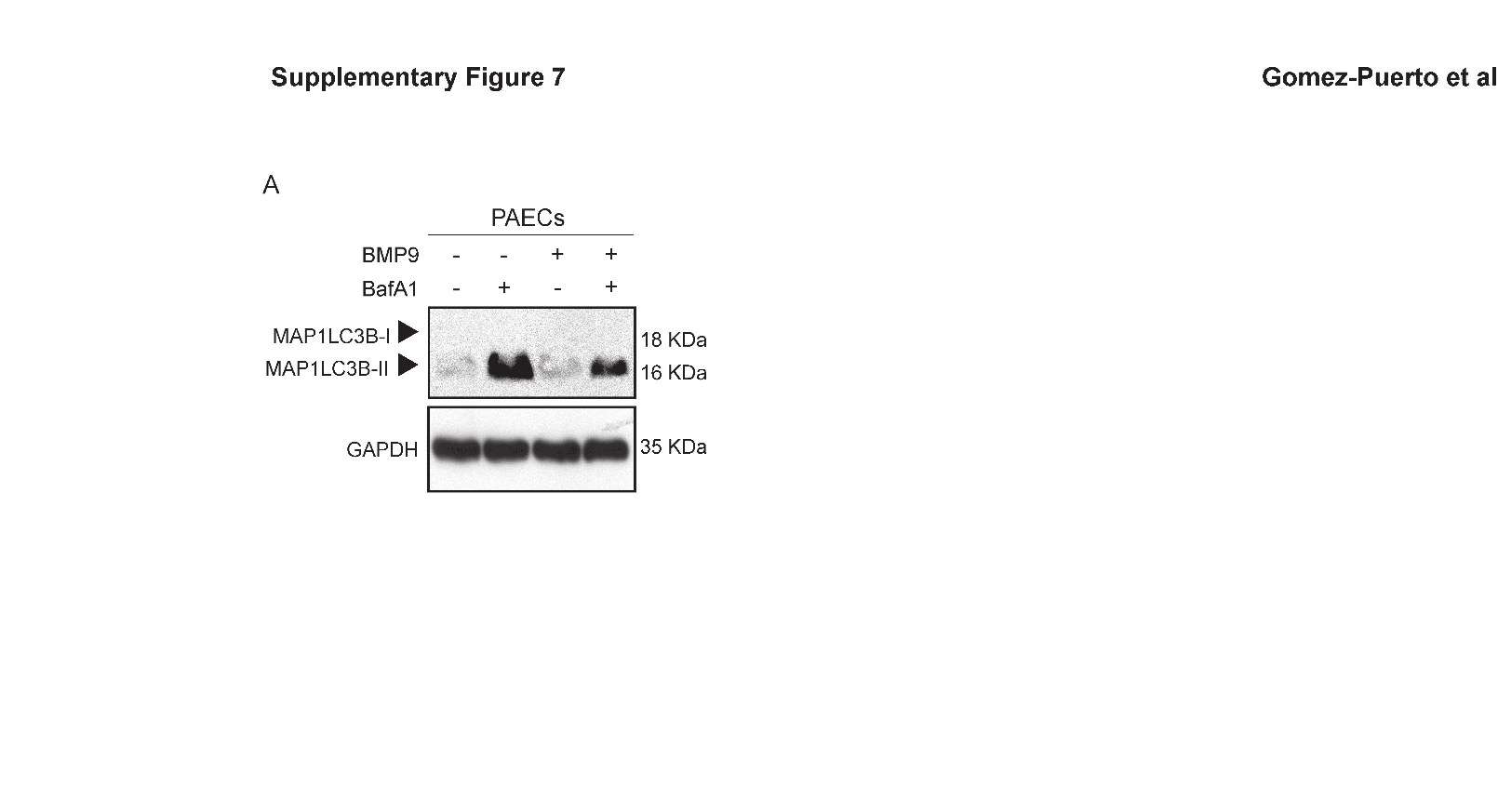
**

**Figure S7.** BMP9 inhibits autophagy in human PAECs**.** (**A**) Western blotting analysis of MAP1LC3B protein expression in PAECs cultured for 16 h in Endothelial Cell Growth Medium MV 2 (without growth factors) supplemented with 0.1% FBS in the presence of BafA1 (20 nm) and BMP9 (1 ng/ml). GAPDH was used as a loading control. Representative results of at least three independent experiments are shown.
