# Supplementary material for: Autophagy contributes to BMP type 2 receptor degradation and development of pulmonary arterial hypertension
Source: J Pathol. 2019 Aug 27;249(3):356–67. doi: 10.1002/path.5322 (PMC6852495; doi:10.1002/path.5322)
Supplement: Supplementary file 2 — Table S1. List of primers used for RT‐qPCR [file PATH-249-356-s002.docx]

**Autophagy contributes to BMP type 2 receptor degradation and development of pulmonary arterial hypertension**

Gomez-Puerto MC *et al*. *J Pathol* DOI: 10.1002/path.5322

**Table S1.** List of primers used for RT-qPCR

| **Gene** | **Forward primer** | **Reverse primer** |
| --- | --- | --- |
| ATG7 | CAGTTTGCCCCTTTTAGTAGTGC | CCTTAATGTCCTTGGGAGCTTCA |
| BMPR2 | AACTGTTGGAGCTGATTGGC | CGGTTTGCAAAGGAAAACAC |
| GAPDH | AGCCACATCGCTCAGACAC | GCCCAATACGACCAAATCC |
